# Supplementary material for: Transcriptional, Epigenetic, and Functional Reprogramming of Monocytes From Non-Human Primates Following Chronic Alcohol Drinking
Source: Front Immunol. 2021 Aug 20;12:724015. doi: 10.3389/fimmu.2021.724015 (PMC8417707; doi:10.3389/fimmu.2021.724015)
Supplement: Supplementary file 6 [file Table_1.docx]

| **Animal ID** | **Sex** | **Mean daily ethanol intake (g/kg/day) ± Standard Deviation** | **Blood**  **Ethanol**  **Content**  **(Avg mg%)** |
| --- | --- | --- | --- |
|  |  |  |  |
| C1 | F | 0 | 0 |
| C2 | F | 0 | 0 |
| C3 | F | 0 | 0 |
| C4 | M | 0 | 0 |
| C5 | M | 0 | 0 |
| CHD1 | F | 3.3 ± 1.0 | 41 |
| CHD2 | F | 3.9 ± 1.0 | 46 |
| CHD3 | F | 4.0 ± 1.1 | 57 |
| CHD4 | F | 4.0 ± 1.3 | 65 |
| CHD5 | F | 5.0 ± 1.4 | 81 |
| CHD6 | F | 5.2 ± 1.2 | 103 |
| CHD7 | M | 2.9 ± 1.3 | 74 |
| CHD8 | M | 3.2 ± 0.9 | 79 |

**Table 1: Summary of samples used in this study.** Mean daily ethanol (EtOH) intake reflects the average dose consumed during the period of 12-month self-administration period.
